# Improved Dissolution and Oral Bioavailability of Valsartan Using a Solidified Supersaturable Self-Microemulsifying Drug Delivery System Containing Gelucire^®^ 44/14

**DOI:** 10.3390/pharmaceutics11020058

**Published:** 2019-01-31

**Authors:** Dong Jun Shin, Bo Ram Chae, Yoon Tae Goo, Ho Yub Yoon, Chang Hyun Kim, Se Il Sohn, Dongho Oh, Ahram Lee, Seh Hyon Song, Young Wook Choi

**Affiliations:** 1 College of Pharmacy, Chung-Ang University, 84 Heuksuk-ro, Dongjak-gu, Seoul 06974, Korea; sdj0130@hanmail.net (D.J.S.); hifyram@daewonpharm.com (B.R.C.); rndbsxo5318@naver.com (Y.T.G.); phantomryda@naver.com (H.Y.Y.); yj.ch.kim@gmail.com (C.H.K.); songsehhyon@hanmail.net (S.H.S.); 2 Daewon Pharm. Co., Ltd, 520 Cheonhodae-ro, Gwangjin-gu, Seoul 04994, Korea; sisohn@daewonpharm.com (S.I.S.); frank82@daewonpharm.com (D.O.); ahramlee@daewonpharm.com (A.L.)

**Keywords:** Valsartan, SMEDDS, Gelucire^®^ 44/14, solidification, bioavailability

## Abstract

To improve the dissolution and oral bioavailability of valsartan (VST), we previously formulated a supersaturable self-microemulsifying drug delivery system (SuSMED) composed of Capmul^®^ MCM (oil), Tween^®^ 80 (surfactant), Transcutol^®^ P (cosurfactant), and Poloxamer 407 (precipitation inhibitor) but encountered a stability problem (Transcutol^®^ P-induced weight loss in storage) after solidification. In the present study, replacing Transcutol^®^ P with Gelucire^®^ 44/14 resulted in a novel SuSMED formulation, wherein the total amount of surfactant/cosurfactant was less than that of the previous formulation. Solidified SuSMED (S-SuSMED) granules were prepared by blending VST-containing SuSMED with selective solid carriers, L-HPC and Florite^®^ PS-10, wherein VST existed in an amorphous state. S-SuSMED tablets fabricated by direct compression with additional excipients were sufficiently stable in terms of drug content and impurity changes after 6 months of storage at accelerated conditions (40 ± 2 °C and 75 ± 5% relative humidity). Consequently, enhanced dissolution was obtained (pH 1.2, 2 h): 6-fold for S-SuSMED granules against raw VST; 2.3-fold for S-SuSMED tablets against Diovan^®^ (reference tablet). S-SuSMED tablets increased oral bioavailability in rats (10 mg/kg VST dose): approximately 177–198% versus raw VST and Diovan^®^. Therefore, VST-loaded S-SuSMED formulations might be good candidates for practical development in the pharmaceutical industry.

## 1. Introduction

Valsartan (VST), a selective blocker for the type I angiotensin II receptor, has been used for the treatment of cardiac diseases [1]. VST belongs to class II of the biopharmaceutical classification system (BCS) and has a low oral bioavailability (BA) because of its limited solubility in water and acidic pH environments [2]. To solve this problem, various techniques including cyclodextrin complexation, solid dispersion, microemulsification, micelles, and nanosuspension have been widely introduced [3,4,5,6]. Among these, an approach using a self-microemulsifying drug delivery system (SMEDDS) composed of oil, surfactant, and cosurfactant has been extensively investigated [7,8]. SMEDDS can form oil-in-water (o/w) microemulsions voluntarily in the gastrointestinal (GI) tract, allowing rapid dissolution and improved permeation of the drug through the intestinal membranes [9].

Gelucire^®^ 44/14 (lauroyl polyoxyl-32 glycerides; GEL) is an inert semi-solid waxy material with a melting point of 44 °C and an HLB value of 14. It was recently introduced as a self-emulsifying non-ionic surfactant, and thus is frequently used in type III lipid-based formulations including SMEDDS [10,11]. This excipient offers broad use flexibility: in addition to filling hard or soft gelatin capsules, it may also be used to make pellets, spheroids, or matrix forms [12,13]. Employing GEL, improvement of the dissolution and BA of numerous water-insoluble drugs has been successfully achieved [11,14]. As GEL contains a well-balanced proportion of short-, medium-, and long-chain fatty acid esters, it forms an exceptionally stable, fine dispersion when in contact with the GI fluids at body temperature. 

Previously, we demonstrated that a supersaturable SMEDDS (SuSMED), consisting of Capmul^®^ MCM (oil), Tween^®^ 80 (surfactant), Transcutol^®^ P (cosurfactant), and Poloxamer 407 (POL; a precipitation inhibitor), greatly contributed to the enhanced dissolution and oral absorption of VST [9,15]. Furthermore, the SuSMED was formulated as solid granules and tablets by employing solid carriers, Florite^®^ PS-10 (FLO) and Vivapur 105 [15]. All prepared formulations revealed enhanced dissolution and oral absorption. However, unexpectedly, during storage in a plastic container with silica desiccant at the ambient condition of 25 °C, we encountered a stability problem (approximately 5% weight loss of tablets after 4 weeks; unpublished data), which might be attributable to the fractional evaporation of Transcutol^®^ P due to low vapor pressure (0.07–0.12 mmHg at 20 °C) [16]. 

Thus, in the present study, by replacing Transcutol^®^ P with GEL, a novel SuSMED was formulated, in which the total amount of surfactant and cosurfactant was reduced. Further, solidified SuSMED (S-SuSMED) granules were prepared by blending VST-containing SuSMED with selective solid carriers, and their solid characteristics were evaluated in terms of the crystalline state and flow property. Furthermore, S-SuSMED tablets were fabricated by direct compression with additional excipients. Consequently, compared to raw VST and Diovan^®^ (a commercial product used as a reference), prepared S-SuSMED granules and tablets exhibited not only increased dissolution in a pH 1.2 medium but also enhanced oral BA in rats.

## 2. Materials and Methods

### 2.1. Materials

VST was supplied by Daewon Pharm. Co., Ltd. (Seoul, Korea). Diovan^®^ tablets containing 80 mg of VST were purchased from a commercial source and used as a reference product. Capmul^®^ MCM (glyceryl caprylate/caprate) was purchased from Abitec Co. (Janesville, WI, USA). FLO (calcium silicate) was purchased from Tomita Pharmaceutical Co., Ltd. (Tokushima, Japan). Capryol 90, GEL, Labrasol^®^, Laurogylcol 90, and Transcutol^®^ P were supplied by Gattefossé (Saint Priest, France). High-performance liquid chromatography (HPLC)-grade acetonitrile and methanol were purchased from JT Baker (Phillipsburg, NJ, USA). Low-substituted hydroxypropyl cellulose B1 (LH-B1; HPC) was purchased from Shin-Etsu Chemical Co., Ltd. (Tokyo, Japan). Magnesium stearate was purchased from FACI (Genoa, Italy). Cremophor^®^ EL, Kollidon^®^ 30 (polyvinylpyrrolidone K30), and POL (Pluronic^®^ F-127) were purchased from BASF (Ludwigshafen, Germany). Primellose^®^ (croscarmellose sodium) was purchased from DFE Pharma (Nörten-Hardenberg, Germany). Tween^®^ 80 was purchased from Sigma-Aldrich (St. Louis, MO, USA). Polyethylene glycol 400 (PEG 400) and propylene glycol were purchased from Duksan Pure Chemical Co., Ltd. (Ansan, Korea). All other chemicals used were of analytical grade.

### 2.2. Animals

Male Sprague Dawley rats, 8 weeks old and 200–250 g weighed, were purchased from Orient Bio (Gyeonggi-do, Korea). Prior to drug administration, all rats were fasted overnight for 12–18 h but freely accessed with water intake. All animal experiments were conducted in compliance with the National Institute of Health (NIH) guidelines “Principles of laboratory animal care” and were approved by the Institutional Animal Care and Use Committee of Chung-Ang University (Protocol No. 2018-00120), Seoul, Korea.

### 2.3. Solubility Test

To screen a suitable oil, surfactant (S), and cosurfactant (CoS) as SMEDDS constituents, equilibrium solubility of VST in various vehicles was measured [9]. Briefly, an excess of VST was added to 1 mL of the selected vehicle. Test tubes containing the mixture were sealed and kept in ambient conditions with intermittent shaking (CM-1000, EYELA, Tokyo, Japan) for 24 h to reach equilibrium. To remove the undissolved VST, the mixtures were then centrifuged (Smart R17, Hanil Science Industrial, Incheon, Korea) at 14,000× *g* for 10 min. The supernatant was filtered through a 0.45-μm polyvinylidene difluoride (PVDF) membrane filter (Whatman International Ltd., Kent, UK), and, after appropriate dilution with methanol, the concentration of VST in the filtrate was quantified using HPLC. 

### 2.4. HPLC Analysis of VST

HPLC analysis was performed to determine the concentration of VST. The HPLC system consisted of a pump (W2690/5; Waters Corporation, Milford, MA, USA), an ultraviolet detector (W2489; Waters Corporation, Milford, MA, USA), and a data station (Empower 3; Waters Corporation, Milford, MA, USA). Using a C18 column (250 × 4.6 mm, 5 μm; Shiseido, Tokyo, Japan), the isocratic mobile phase, composed of distilled water and acetonitrile (40:60 [*v*/*v*]) and pH-adjusted to 3.0 by adding 10% phosphoric acid, was eluted at a flow rate of 1.0 mL/min at 25 °C. Finally, aliquots (20 μL) of each sample were injected into the column, and the absorbance was measured with ultraviolet detection at 247 nm. The calibration curve was obtained by least-square linear regression in the range 10–120 μg/mL, with a coefficient determination (*R*^2^) value of >0.99. 

### 2.5. Construction of Pseudo-Ternary Phase Diagram

Based on the solubility study, Capmul^®^ MCM, Tween^®^ 80, and Gelucire^®^ 44/14 were selected as an oil, S, and CoS, respectively. The ternary phase diagram composed of water, oil, and S/CoS was constructed using a water titration method in a drug-free environment [9]. While the blended ratios of S/CoS were maintained as 1:2, 1:1, and 2:1 (*v*/*v*), the proportion of the oil component in the S/CoS blend varied from 9:1 to 1:9 (*v*/*v*). Then, under gentle stirring, a water droplet was added dropwise into the mixture, with which visual examination was performed to find microemulsion regions of transparent and/or bluish white appearance.

### 2.6. Droplet Size Determination

A dynamic light-scattering particle size analyzer (Zetasizer Nano-ZS, Marlvern Instruments, Worcestershire, UK) was used to measure the droplet size of the microemulsion [9]. The definite volume (10 μL) of each SMEDDS was diluted with 10 mL of distilled water and gently vortexed for 1 min to obtain a homogeneous dispersion. The samples were loaded into a disposable cuvette, placed in a thermostatic chamber at 25 °C, and light scattering was monitored with a 50-mV laser at a 900° angle.

### 2.7. Preparation of VST-Containing SuSMED

Based on the ternary phase diagram and characteristics, the SMEDDS formulation was prepared by mixing 10% Capmul^®^ MCM (oil), 30% Tween^®^ 80 (S), and 60% GEL (CoS). After mixing the SMEDDS components, POL was added as 10% (*w*/*w*) of the SMEDDS components to prepare the SuSMED. The unit dose of VST (80 mg) was added to different quantities (88, 99, 110, 132, 176, and 220 mg) of SuSMED. The components were mixed by vortexing them at 60 °C until the VST was dissolved. In vitro dissolution of the prepared formulations was compared to determine the appropriate composition, and the selected VST-containing SuSMED was subjected to further studies.

### 2.8. In Vitro Dissolution Test

In vitro dissolution tests were performed according to the USP apparatus II (paddle) method with a Vision Classic 6^TM^ dissolution tester and a Vision heater (Hanson, Chatsworth, CA, USA) at 37 ± 0.5 °C. A pH 1.2 medium was made by adding 2 g of sodium chloride to 7 mL of hydrochloric acid and diluting the solution with 1000 mL of distilled water. Each formulation of 80 mg VST equivalent was introduced into 900 mL of pH 1.2 medium with a revolution speed of 50 rpm. Aliquots (5 mL) were withdrawn at predetermined time points (5, 15, 30, 60, 90, and 120 min) and filtered through a 0.45-μm PVDF membrane filter. An equivalent volume (5 mL) of fresh pH 1.2 medium was replenished to maintain a constant in vitro dissolution environment. The amount of dissolved VST in the filtrate was measured using HPLC after appropriate dilution with methanol.

### 2.9. S-SuSMED Granule Formulation and Characterization

#### 2.9.1. Preparation of VST-Loaded S-SuSMED Granules

Based on previous reports [9,17], two types of solid carriers (HPC and FLO) were selected for the solidification process. S-SuSMED granules were prepared by mixing VST-containing SuSMED (190 mg) with different amounts of HPC/FLO powder blends (ranging from a 0.8-fold to a 1.6-fold amount of SuSMED), in which the HPC and FLO were pre-mixed at a 1:1 (*w*/*w*) ratio. For comparison, granules with different ratios of HPC/FLO were prepared separately, while the total amount of the HPC/FLO blend was fixed at 1.4-fold. After the kneading process, the wet mass was granulated through an 18-mesh sieve and dried in an oven at 60 °C for 12 h. Then, the granules were passed through the 30-mesh sieve and stored at 25 °C in an airtight container. 

#### 2.9.2. Reconstitution Study

A reconstitution study was performed based on an earlier study [15]. The S-SuSMED granules (equivalent to 80 mg of VST) was dispersed in 250 mL of distilled water (DW) by vortex mixing (EYELA, Cute Mixer, CM-1000, Tokyo, Japan) for 1 min, then centrifuged (Micro 17TP; Hanil Science, Incheon, Korea) at 16,000× *g* for 10 min to remove the water-insoluble solids. The size of dispersed droplets in the supernatant was determined as described above. 

#### 2.9.3. Flow Property Observation

The flowability of S-SuSMED granules was evaluated by measuring Carr’s compressibility index (CI) and angle of repose. The CI values were calculated using the equation: CI (%) = 100 × (tapped density − bulk density)/tapped density [18]. The apparent bulk and tapped densities were measured using the cylinder method, with a powder tester (ABD-100, Tsutsui Scientific Instruments Co., Ltd., Tokyo, Japan): the weighed granules were poured into a 50-mL glass cylinder, and the filled cylinder was tapped 100 times by a powder tester until no further reduction in the volume was observed. The angle of repose was determined by pouring the sample through a funnel onto a flat surface and measuring the angle between the horizontal and the slope of the heap of powder [19].

#### 2.9.4. Solid-State Property Assessment

The solid-state properties of the VST, HPC, FLO, physical mixture (PM) of VST and solid carriers, and S-SuSMED granules were investigated using and scanning electron microscopy (SEM), differential scanning calorimetry (DSC), and powder X-ray diffractometry (PXRD). The morphological features of the samples were visualized using a scanning electron microscope (Sigma 300; Carl Zeiss Meditec AG, Jena, Germany). Each powder was fixed on to a brass specimen club using double-sided adhesive tape and coated with platinum using a Hitachi ion sputter (E-1030) for 120 s at 4 mA. The samples were scanned at a voltage of 5 kV. Using DSC-Q20 (TA Instruments, New Castle, DE, USA), DSC thermograms were taken: each sample (3–5 mg) was placed in an aluminum pan and heated at a rate of 5 °C/min for a temperature range from 0–300 °C under nitrogen flow (20 mL/min). Using an X-ray diffractometer (D8 Advance, Bruker, Germany) with nickel-filtered Cu Kα radiation, the PXRD diffractogram was scanned at the 2θ range of 5°–60° with a scanning speed of 5°/min and a step angle of 0.02°.

### 2.10. S-SuSMED Tablet Formulation and Characterization 

#### 2.10.1. Preparation of S-SuSMED Tablets

S-SuSMED granules were homogeneously mixed with excipients of Primellose^®^ (superdisintegrant), magnesium stearate (lubricant), and Kollidon^®^ 30 (binder) using a cube mixer (Type AR400ES, Erweka^®^ GmbH, Heusenstamm, Germany) for 10 min. Using a single-punch tablet press (HANDTAB-200, Ichihashi-Seiki Co. Ltd., Kyoto, Japan), the blended mass was directly compressed into the tablets (compression force of 500 kgf; 12-mm standard circular concave punch). The composition of resultant S-SuSMED tablets, designated as S-SuSMED-T1 and S-SuSMED-T2, were stored in an airtight container at an ambient temperature of 25 °C. 

#### 2.10.2. Characterization of S-SuSMED Tablets

The characteristics of the S-SuSMED tablets were evaluated with regard to weight variation, diameter, thickness, hardness, friability, and drug content. The weight of each tablet was measured using a balance (XS603S analytical balance; Mettler-Toledo, Columbus, OH, USA). The hardness of each tablet was evaluated using a hardness tester (Smart-Test 50; Pharmatron, Solothun, Switzerland). Tablet diameter and thickness were measured using a caliper (ABS Digimatic Caliper; Mitutoyo, Japan). A friability test was performed using a friabilator (FRV1000; Copley scientific, Nottingham, UK): 10 accurately weighed tablets were placed in the friabilator and rotated for 4 min at 25 rpm, and then the tablets were clearly dedusted and accurately re-weighed. 

#### 2.10.3. Drug Content Assay

The S-SuSMED tablets were dispersed in 10 mL of methanol, mixed, and sonicated for 30 min with a bath-type sonicator (Model 2210, Branson Ultrasonics Co., Danbury, CT, USA). After filtering the mixture through a 0.45-μm PVDF membrane filter, the filtrate was diluted with methanol and subjected for HPLC analysis. The drug contents were expressed as percentages of the theoretical quantity.

#### 2.10.4. Stability Assessment

According to the ICH guidelines, stability tests were performed under accelerated and long-term storage conditions in environmental chambers (3949, Thermo Scientific, MA, USA). Test samples of S-SuSMED tablets packed into high-density polyethylene (HDPE) bottles were stored over 6 months at either 40 ± 2 °C and 75 ± 5% relative humidity (RH) for accelerated stability or 25 ± 2 °C and 60 ± 5% RH for long-term storage stability. According to the USP monographs, drug content was determined as described above, and individual impurities in the tablet samples were evaluated as follows: individual impurity = (*R*_u_/*R*_s_) × (*C*_s_/*C*_u_) × 100, where *R*_u_ is the peak response of each impurity from the sample solution, *R*_s_ is the peak response of VST from the standard solution, *C*_s_ is the concentration of USP VST reference standard in the standard solution, and *C*_u_ is the nominal concentration of VST in the sample solution. The concentration below the quantification limit (BQL) range was ≤0.05%. Tablet weights were monitored for significant changes. Separately, after crushing the tablets, reconstitution study was conducted with the same method described above. 

### 2.11. In Vivo Oral Absorption Study

#### 2.11.1. Oral Administration and Plasma Sampling

The rats were randomly divided into four groups (*n* = 8–12): Group 1 received raw VST, Group 2 received Diovan^®^ (powder), Group 3 received S-SuSMED-T1 (powder), and Group 4 received S-SuSMED-T2 (powder). To facilitate ease of administration, the tablet forms of Diovan^®^ and S-SuSMED were ground into powder form and administered using a Torpac^®^ Kit. In all formulations, a dose of 10 mg/kg equivalent to VST was accurately weighed, filled into hard gelatin capsules (Torpac^®^ capsule size 9) (Torpac, Fairfield, NJ, USA) by means of a stand, funnel, and tamper (Torpac^®^ Kit, Torpac), then administered directly into the stomach using a dosing syringe plunger (Torpac^®^ Kit, Torpac). Heparinized capillary tubes were used for blood sample collection from the retro-orbital plexus at predetermined time points (0.33, 0.67, 1, 1.5, 2, 4, 7, 12, and 24 h). After centrifugation of blood samples at 16,000× *g* for 20 min, the plasma samples were stored in light-protected container at −80 °C until the time of analysis by liquid chromatography-tandem mass spectrometry (LC-MS/MS).

#### 2.11.2. VST Determination by LC-MS/MS

Whole plasma samples (50 μL) were mixed with methanol (700 μL) and internal standard (IS) solution (10,000 ng/mL VST-d3 in 50% methanol; 20 μL) and vortexed for 3 min. After centrifugation at 16,000× *g* for 5 min, the supernatant (20 μL) was carefully transferred to a test tube and evaporated to dryness under nitrogen. The dried residue was reconstituted in DW (480 μL), and the mixture was vortexed and centrifuged at 16,000× *g* for 5 min. The supernatant (100 μL) was injected into LC-MS/MS system consisting of an API 4500 triple quadrupole mass spectrometer (Applied Biosystems/MDS SCIEX, Foster City, CA, USA), an Agilent 1260 autosampler (Agilent Technologies Inc, Santa Clara, CA, USA), and a Waters Atlantis dC18 column (50 × 2.1 mm, 3 μm; Milford, MA, USA). An isocratic mobile phase consisting of methanol and 10 mM of ammonium formate (pH 2.7) (80:20, [*v*/*v*]) was used at a flow rate of 0.3 mL/min. Using nitrogen as the collision gas, multiple reaction monitoring mode with positive ion was used for quantitation: the source temperature and ion spray voltage were set at 550 °C and 5.5 kV, respectively. The analytes were detected by monitoring the transitions 436.2 (Q1) → 291.0 (Q3) and 439.2 (Q1) → 294.0 (Q3) m/z for VST and IS, respectively, with a de-clustering potential of 28 V and collision energies of 23 V. The temperature of the nebulizer gas (Gas 1) was 40 °C and that of the heater gas (Gas 2) was 70 °C. For quantifying VST in the plasma samples, each peak area of VST was divided by that of the IS, and the ratio was compared with a calibration curve obtained using a VST standard solution in the same manner. The lower limit of quantification (LLOQ) was 0.05 μg/mL. For concentration values below LLOQ, a value of zero was used in the calculation of pharmacokinetic parameters. 

#### 2.11.3. Pharmacokinetic Assessment

By the linear trapezoidal rule, the area under the curve (AUC) from 0 to 24 h was calculated using the BA Calc 2007 pharmacokinetic analysis program (Ministry of Food and Drug Safety, Chungcheongbuk-do, Korea). The *C*_max_ (maximum plasma concentration) and *T*_max_ (the time to reach the *C*_max_) were determined directly from the concentration-time data. Relative BA (RBA) was calculated by dividing the AUC of the test samples with those of the Diovan^®^.

### 2.12. Statistical Analysis

All data are represented as mean ± standard deviation (SD). Statistical significance was evaluated using the Student’s *t*-test, with *p* < 0.05 considered statistically significant.

## 3. Results and Discussion

### 3.1. Screening of SMEDDS Components

**Figure 1:**
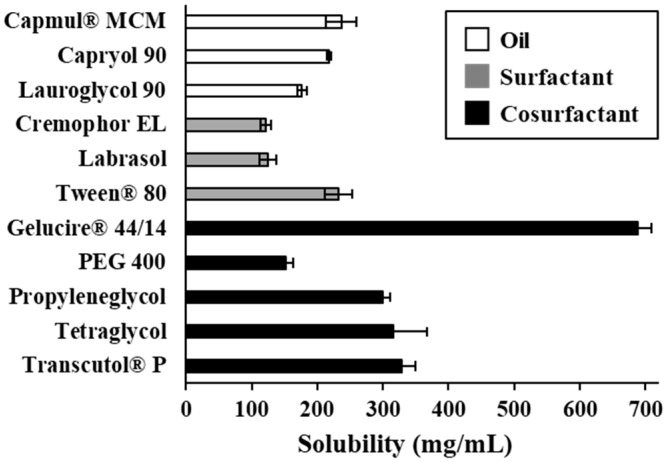
Solubility of valsartan (VST) in various vehicles for self-microemulsifying drug delivery system (SMEDDS) formulation. Values are presented as the mean ± SD (*n* = 3).

It is crucial to screen SMEDDS components with high drug-solubilizing capacities for successful formulations. The oil plays a key role in solubilizing hydrophobic drugs, and the S/CoS helps to form a stable nanodispersion [9]. The solubility of VST in various vehicles is shown in Figure 1. Among the oil components, Capmul^®^ MCM showed the greatest solubility (236.6 ± 23.8 mg/mL) compared with Capryol^®^ 90 or Lauroglycol^®^ 90. Tween^®^ 80 was selected as a surfactant because of greater solubility (232.6 ± 20.9 mg/mL) than that of Cremophor^®^ EL or Labrasol^®^. Interestingly, GEL exhibited a greatly increased solubility (688.7 ± 20.7 mg/mL), which was over 2-fold greater than that of other cosurfactants (152.5–328.2 mg/mL on average). Thus, for a SMEDDS formulation, Capmul^®^ MCM, Tween^®^ 80, and GEL were selected as oil, S, and CoS, respectively. Specifically, Tween^®^ 80 and GEL are well-known P-glycoprotein (P-gp) inhibitors [20,21]. In this aspect, since VST is a P-gp substrate, concomitant action of these components for further BA enhancement would be expected [21].

### 3.2. Construction of Pseudo-Ternary Phase Diagram

**Figure 2:**
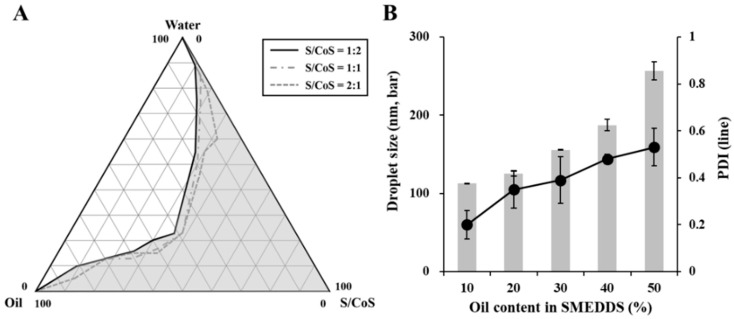
(**A**) Pseudo-ternary phase diagram composed of water, Capmul^®^ MCM (oil), and surfactant mix composed of Tween^®^ 80 (surfactant, S), and Gelucire^®^ 44/14 (GEL) (cosurfactant, CoS); (**B**) Effect of oil content in SMEDDS (S/CoS = 1:1) on droplet size (nm, bar) and polydispersity index (PDI; line). Values are presented as the mean ± SD (*n* = 3). Light gray area in Figure 2A indicates the region for self-microemulsification with transparent or clear bluish-white appearance.

The pseudo-ternary phase diagram was constructed to determine the proper S/CoS ratio and to find a self-microemulsification region. As shown in Figure 2A, the light gray area indicates the self-microemulsification region with a transparent or clear bluish-white appearance. The broader self-microemulsification region was obtained when the S/CoS ratio was 1:2, but the difference was not significant. The microemulsification area varied as the proportion of oil in the SMEDDS changed. To find a proper composition, the effect of oil content on the SMEDDS (S/CoS ratio of 1:2) was evaluated based on droplet size and polydispersity index (PDI) values (Figure 2B). The droplet size increased as the oil content increased. The smallest droplet size (112.7 ± 0.2 nm) with a PDI value of 0.2 was obtained in 10% oil content. The small droplet size of the SMEDDS with homogeneity provided enhanced dissolution and absorption due to increased surface area [22]. Thus, to prepare the SMEDDS, the optimal ratio of oil/S/CoS was fixed as 1:3:6 (*w*/*w*/*w*) and used for further studies.

### 3.3. Formulation of VST-Containing SuSMED

**Figure 3:**
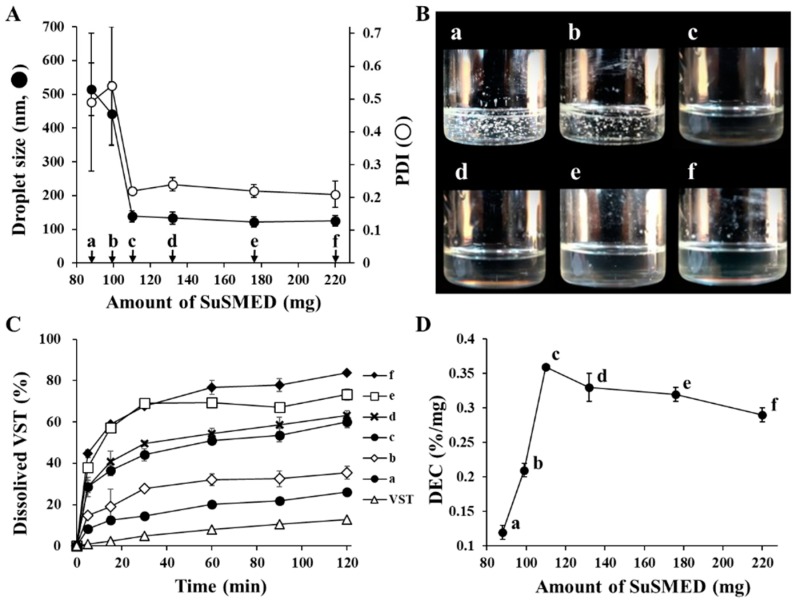
Drug loading capacity of the supersaturable self-microemulsifying drug delivery system (SuSMED) expressed as the added amount of the SuSMED containing the unit dose of VST 80 mg. Values are presented as the mean ± SD (*n* = 3). (**A**) Changes in droplet size and PDI values; (**B**) visual observation of dispersion behavior; (**C**) dissolution profiles for 2 h; (**D**) comparison with dissolution enhancing capacity (DEC).

Based on a previous study [9], the SuSMED was prepared by adding POL (10% in weight basis) to the SMEDDS formulation as a supersaturating agent. With this formula, to solubilize VST 80 mg, a recommended unit dose per oral administration, the minimum required amount of SuSMED was investigated. As shown in Figure 3, VST 80 mg was added to different amounts of SuSMED (88, 99, 110, 132, 176, and 220 mg, designated as compositions a, b, c, d, e, and f, respectively), and the drug-loading capacity of SuSMED was characterized in terms of droplet size changes, dispersion behavior, and dissolution performances. Figure 3A depicts that compositions a and b showed large droplet sizes with no homogeneity, while compositions c–f showed small droplet sizes (121.2–138.8 nm) with homogeneity (PDI of 0.21–0.22). This dispersion behavior was further visualized by naked eye observation (Figure 3B). As expected, a and b did not completely solubilize the VST added, whereas other compositions totally solubilized the unit dose of VST, resulting in a clear and transparent appearance. In addition, the dissolution behavior of all compositions and the VST powder was evaluated. As shown in Figure 3C, VST powder exhibited poor dissolution, with approximately 10% dissolution in 2 h. In contrast, all compositions (a–f) showed an enhancement in dissolution. However, dissolution of compositions a and b reached a relatively low level (less than 35% for 2 h) compared with other compositions that achieved a rapid increase with high-level dissolution (60–84% for 2 h). For further comparison, the dissolution efficiency (DE) and dissolution enhancing capacity (DEC) were calculated as follows: DE (%) = $\left[\int_{t1}^{t2}y\mathrm{dt}/y_{100}\cdot \left(t2-t1\right)\right]\times 100$, where *y* is the percentage of dissolved product [23]; DEC = (DE_SuSMED_ − DE_VST_)/W_SuSMED_, where DE_SuSMED_ and DE_VST_ are the DE of the VST-loaded SuSMED formulation and VST, respectively, and W_SuSMED_ is the amount (mg) of SuSMED [9]. As shown in Figure 3D, composition c showed the greatest DEC value, indicating its high efficiency of VST dissolution on a unit weight basis. Afterward, DEC values gradually decreased as the amount of SuSMED increased. Based on these results, we finally selected composition c, consisting of VST 80 mg, SMEDDS 100 mg, and POL 10 mg, for further granulation studies. This GEL-based composition contains approximately 20% less S/CoS than the Transcutol^®^ P-based SuSMED reported previously [15]. 

### 3.4. Formulation of S-SuSMED Granules

#### 3.4.1. Selection of Solid Carriers and Granulation

The solid carrier selection is one of the most important factors for developing solidified formulations of the liquid-state SMEDDS. Solidification offers several advantages, including feasibility of manufacturing and the stabilization of SMEDDS, while getting rid of liquid-induced processing limitations [24]. There are several types of solid carriers with various properties, such as pore size, oil-absorption capacity, desorption properties, water-soluble properties, surface areas and particle size, that affect the solidification of SMEDDSs [15]. Typically, water-insoluble carriers (FLO, Neusilin^®^ US2, Aerosil^®^ 200, and Sylysia^®^ 350) and cellulose-based hydrophilic excipients (HPC, Vivapur^®^ 105, and Avicel^®^ PH101) have been used [15,25,26,27]. In this study, FLO and HPC were selected as solid carriers for preparing S-SuSMED. FLO, a silica-based adsorbent with a high oil-absorbing capacity (4.6 mL/g) [28], is frequently used as an adsorbent in the solidification process of liquid formulations [29], since it provides a sufficiently large surface area and large volume of macrospores for liquid absorption [28]. Moreover, it showed the highest dissolution efficiency and SMEDDS-absorbing capacity for reducing the number of solid carriers among the screened adsorbents: Sylysia^®^ 350, Neusilin^®^ US2, and FLO [9,30]. However, despite the above merits, FLO shows an incomplete desorption of SMEDDS components due to hydrophobic interactions and/or van der Waals forces between the hydroxyl group of VST and the silanol moiety on the surface of the silica-based adsorbent [24,31]. Therefore, the combination of a hydrophilic diluent with the property of high desorption was necessary. HPC is a water-absorbable diluent commonly used as a disintegrant and/or binder for direct compression [17,27]. The enhancing effects of HPC on drug release and disintegration were reported following a swelling process through water absorption and penetration [32]. In addition, HPC was introduced as a diluent for direct compression of fluoxetine tablets and selected as a diluent and disintegrant due to its binder properties for the invention of dipeptidyl peptidase IV inhibitor formulations [33,34].

**Figure 4:**
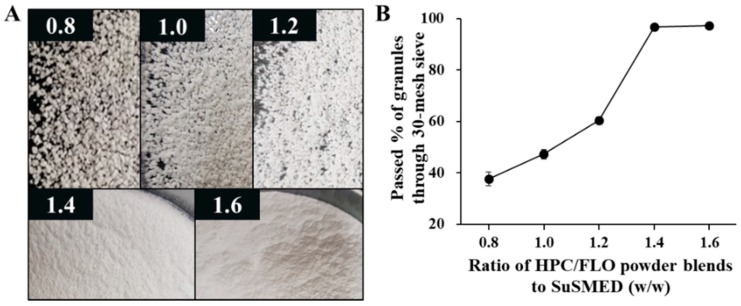
Screening of a proper quantity of solid carriers (hydroxypropyl cellulose (HPC)/Florite^®^ PS-10 (FLO) blends) for granulation. Different quantities of solid carriers (ranging from 0.8-fold to 1.6-fold the amount of SuSMED) were mixed with a definite amount of VST-containing SuSMED (190 mg). (**A**) The appearance of the granulated mass before drying (no magnification); (**B**) percentage of granules passed through a 30-mesh sieve against the quantity of solid carriers. Values are presented as the mean ± SD (*n* = 3).

With the combination of HPC and FLO, a preliminary study was performed to determine the optimal amount of solid carrier for the granulation. Different amounts of the HPC/FLO powder blend (ranging from 0.8-fold to 1.6-fold the amount of the SuSMED) were mixed with a definite amount of VST-containing SuSMED (190 mg in total). As shown in photographs (Figure 4A), depending on the amount of the HPC/FLO powder blend added, the appearance of the granulated mass before drying was different: coarse and aggregated particles appeared with a smaller amount of the powder blend, while fine and free-flowing particles occurred with a larger amount. For further comparison, the percentage of granules passed through a 30-mesh sieve was plotted against the amount of the HPC/FLO powder blend added (Figure 4B). As the amount of the powder blend added increased, the passed % increased proportionally, up to the level of 1.4-fold the amount of powder blend. Beyond this amount, no difference was observed. Therefore, the amount of HPC/FLO powder in the blends was fixed at 1.4-fold, representing the addition of 266 mg of powder to the VST-containing SuSMED (190 mg) for granulation.

#### 3.4.2. Physical Characteristics of Various S-SuSMED Granules

While the amount of the HPC/FLO powder blend was fixed at 266 mg, different S-SuSMED granules were prepared by varying the ratio of HPC and FLO. As listed in Table 1, based on the amount of VST-containing SuSMED (190 mg), HPC and FLO (1.4-fold in total) were added at different *w*/*w* ratios of 0.1:1.3 (G1), 0.3:1.1 (G2), 0.5:0.9 (G3), 0.7:0.7 (G4), 0.9:0.5 (G5), and 1.1:0.3 (G6). Granulation did not affect the microemulsion-forming capacity of the SuSMED. Upon reconstitution, all prepared granules revealed a droplet size in the range of 136.0–151.2 nm with PDI values of 0.23–0.34, indicating a stable and homogeneous nanodispersion [26].

**Figure 5:**
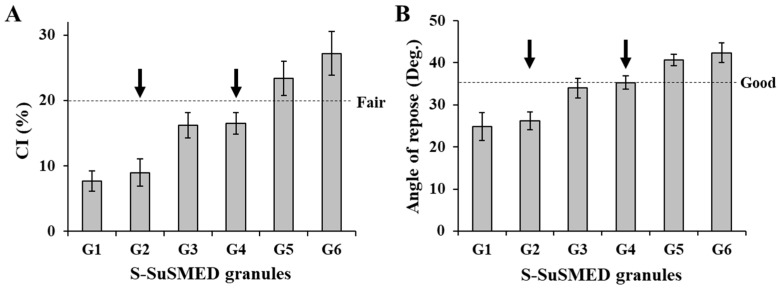
Flowability comparison of different S-SuSMED granules. Arrows indicate the selected formulae for tablet preparation. Values are presented as the mean ± SD (*n* = 3). (**A**) Carr’s compressibility index (CI); (**B**) angle of repose.

On the other hand, the flow property of S-SuSMED granules was compared in terms of CI and angle of repose. According to the USP guidelines, flowability in terms of the CI value was classified as excellent (1–10), good (11–15), fair (16–20), passable (21–25), and poor (>26). As shown in Figure 5A, G1 and G2 granules revealed CI values of 7.7% and 9.0%, respectively, indicating excellent flowability. G3 (16.2%) and G4 (16.5%) granules were graded as fair, while G5 (23.4%) and G6 (27.2%) granules were categorized as passable to poor. Similar trends were observed in the comparison of angles of repose (Figure 5B): G1 and G2 granules showed less than 30°, indicating excellent flowability; G3 and G4 granules were graded as good, while G5 and G6 granules were graded as passable. Flowability improved as the amount of FLO increased, which might be attributable to its high oil-absorbing capacity (4.6 mL/g) [28]. In contrast, as the ratio of HPC in the mixed solid carriers increased, the granules became aggregated to form a sticky mass, since HPC is a cellulose-based diluent with hydrophilic and viscous properties [17]. Overall, G1–G4 granules revealed acceptable flowability. Among them, based on HPC/FLO ratio, G2 and G4 granules were ultimately selected as the representative formulae for tablet preparation. If the flowability were similar, a high portion of HPC is expected to possess greater dissolution because of its water-soluble property.

#### 3.4.3. Solid-State Property Assessment

**Figure 6:**
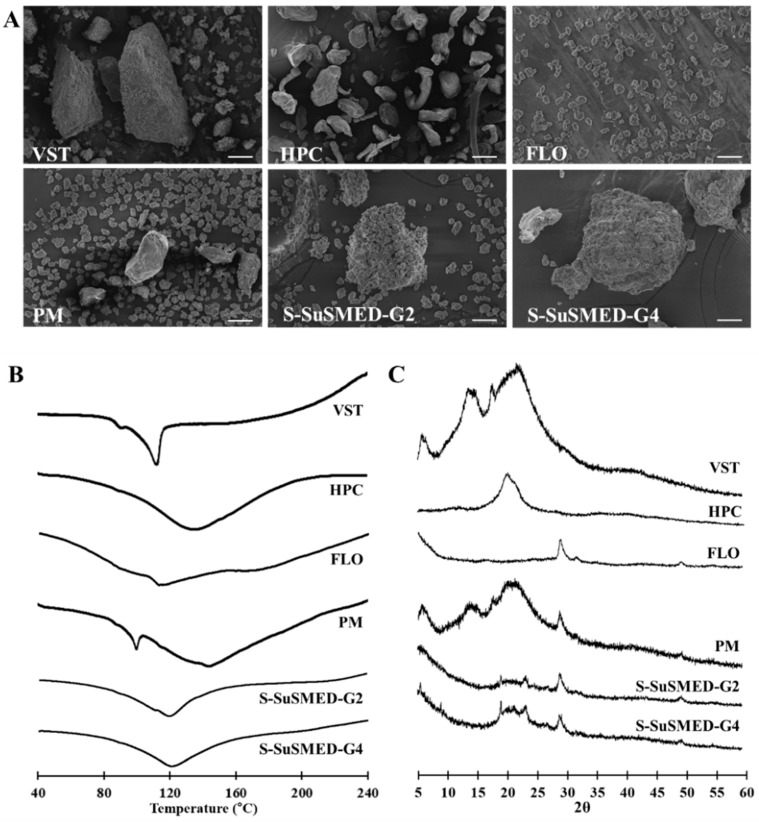
Solid-state properties of different samples: VST, HPC, FLO, and physical mixture (PM) of VST and solid carriers; S-SuSMED-G2; and S-SuSMED-G4: (**A**) scanning electron microscopy (SEM) images. scale bar = 30 μm; (**B**) differential scanning calorimetry (DSC); (**C**) powder X-ray diffractometry (PXRD).

To evaluate the effects of adsorption into the solid carriers on the crystalline structure of the active pharmaceutical ingredient, different samples (VST, HPC, FLO, and PM of VST and solid carriers; S-SuSMED-G2; and S-SuSMED-G4) were subjected to SEM, DSC, and PXRD analyses. As shown in Figure 6A, SEM reveled the morphologies of various solid samples. VST as a raw material appeared to have a rectangular-shaped crystalline structure. No distinct crystals of VST were observed in both G2 and G4 granules but were shown in PM of VST and solid carriers, indicating that VST was solubilized and absorbed onto the solid carriers. DSC thermograms are shown in Figure 6B. Thermal analysis can provide information related to melting, recrystallization, decomposition, and changes in the specific heat capacity, which determines the physicochemical properties of a compound. The DSC curve for raw VST powder exhibited a sharp endothermic peak at 112.2 °C, which corresponds to its intrinsic melting point (115–116 °C) [35]. There were no specific endothermic peaks for both solid carriers, consistent with the previously reported DSC thermograms [15]. The PM of VST and solid carriers showed a mixed pattern for each component: a small sharp peak around 100 °C, which possibly originated from the VST peak, and a broad endothermic peak between 110 °C and 150 °C. However, no melting peak of VST was identified in either of the S-SuSMED granules, indicating that VST was completely solubilized in SuSMED and adsorbed into the solid carriers, existing in a non-crystalline state. Simultaneously, it could be possible that the endothermic peak of VST had decreased due to low VST content in the sample: the final drug content (*w*/*w*) of PM and S-SuSMED granules were 23.1% and 17.5%, respectively. Thus, to further investigate the crystallinity of VST in S-SuSMED, PXRD analysis was conducted. PXRD patterns of the same samples are presented in Figure 6C. The diffraction pattern of VST showed various strong broad peaks at 6.9°, 13.8°, and 21.9°, indicating its crystalline nature as previously reported [36]. HPC showed a huge intrinsic peak at 20.1°, and FLO showed its intrinsic peak at 28.0°. PM showed all major characteristic peaks of VST and solid carriers with slightly reduced intensity at various angles, indicating that the crystalline nature of the VST was maintained. In comparison, the peak pattern of both S-SuSMED granules was similar, revealing no specific intrinsic peaks of VST. These results suggested that the internal structural transition of VST existed in an amorphous state and/or was totally solubilized in the SuSMED components.

### 3.5. Formulation of S-SuSMED Tablets

#### 3.5.1. S-SuSMED Tablet Preparation

S-SuSMED tablets were prepared by direct compression of the blended mixture of S-SuSMED granules and other excipients such as Kollidon^®^ 30 (binder), Primellose^®^ (superdisintegrant), and magnesium stearate (lubricant). The compositions and physical characteristics of the S-SuSMED tablets are listed in Table 2. Kollidon^®^ 30 is widely used as a directly compressible binder at a 2–10% (*w*/*w*) ratio [37,38]. In this study, it was added at a 10% (*w*/*w*) ratio to provide sufficient binding capacity. However, the tablets were not disintegrated until 60 min, resulting in poor VST dissolution (data not shown). Thus, to accelerate the tablet disintegration, Primellose^®^ was added at a 5% (*w*/*w*) ratio. This excipient is an excellent disintegrating agent, due to its mixed mechanisms of swelling, wicking, and strain recovery [39], and it has been used in the range of 3–9% (*w*/*w*) ratios [15,19,40]. In addition, magnesium stearate was used as a lubricant at a 2% (*w*/*w*) ratio to avoid the processing problem of sticking and weight variation. As a result, the weights of the S-SuSMED-T1 and S-SuSMED-T2 were identical, indicating no weight loss during the compression process. Both tablets showed similar drug contents of over 97%. In the aspect of tablet dimension, the diameter was the same at approximately 12 mm, but the thickness was different. The thickness of S-SuSMED-T1 (4.83 mm) was greater than that of S-SuSMED-T2 (4.31 mm). This difference might be due to the high FLO content of the former, because it has a lower bulk and tapped density (0.08 and 0.12 g/mL, respectively) compared with HPC (0.5 and 0.7 g/mL, respectively) [17,28]. However, the hardness was not different between the two tablets, revealing a friability of less than 1% for both tablets.

#### 3.5.2. Stability of S-SuSMED Tablets

The result of the study of accelerated and long-term stability conditions are shown in Table 3. There was no significant change in the drug content of Diovan^®^, S-SuSMED-T1, and S-SuSMED-T2 after being placed in storage for 6 months. The unknown impurity peaks of S-SuSMED-T1 and S-SuSMED-T2 were detected as BQL (≤0.05%) at all tested periods. However, 0.06% of unknown impurity peaks were measured in Diovan^®^ after 6 months in an accelerated condition. Thus, we believe that the S-SuSMED tablets are stable enough in terms of drug content and impurity changes. In addition, the weights of the tablets remained constant for 6 months, and the droplet size on reconstitution study exhibited the same range (130–150 nm in average) as initially obtained, demonstrating their excellent physical stability due to the advantage afforded by replacing Transcutol^®^ P with GEL. 

### 3.6. In Vitro Dissolution Profiles of S-SuSMED Granules and Tablets

**Figure 7:**
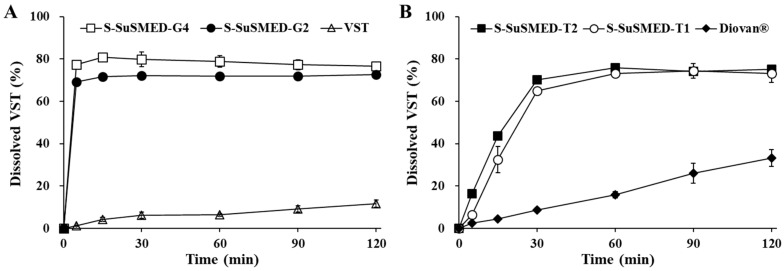
In vitro dissolution profiles of VST-containing granules (**A**) and tablets (**B**) in a pH 1.2 medium. VST powder and Diovan^®^ tablets were used as references for comparison. Values are presented as the mean ± SD (*n* = 3).

The dissolution of VST is pH-dependent, exhibiting approximately 100% drug release in a pH 6.8 phosphate buffer as compared with very low dissolution in a pH 1.2 buffer, due to its extremely low solubility in acidic pHs [2,7,9]. In this study, the dissolution profiles of various samples, including raw VST, Diovan^®^, S-SuSMED granules, and S-SuSMED tablets, were evaluated in a pH 1.2 medium for a 2 h period. As shown in Figure 7A, the dissolution level of raw VST was lower (reaching approximately 12% at 2 h) than that of the S-SuSMED granules (over 75% at 2 h). Compared with the gradual increase in raw VST, the S-SuSMED granules showed rapid dissolution (more than 70% in 5 min). Although S-SuSMED-G4 exhibited slightly higher dissolution than S-SuSMED-G2, their overall behaviors were similar. Meanwhile, the dissolution profiles of tablet formulations are compared in Figure 7B. Diovan^®^ gradually released the drug, resulting in a dissolution level of approximately 33% at 2 h. In contrast, the S-SuSMED tablets showed significantly enhanced VST dissolution. The dissolution patterns of both S-SuSMED-T1 and S-SuSMED-T2 were similar, showing a biphasic release pattern (a gradual increase over 30 min and a plateau after 1 h). The dissolution outcomes for the tablets in the earlier stages were different from those of the granules, representing the time required for tablet disintegration. However, for both granules and tablets, the degree of dissolution at the final stage remained the same. Thus, it is possible to propose that the tablets disintegrate first, then the components of SuSMED rapidly desorb from the solid carriers to form nano-sized homogeneous emulsions. Owing to the homogeneously dispersed oil droplets, in which the drug exists in molecular solubilization, pertinent dissolution could take place. This behavior was consistent with our earlier report that Transcutol^®^ P-employed S-SuSMED granules and tablets showed a rapid release and a gradually increased release, respectively [15]. Compared with raw VST and Diovan^®^, the earlier formulation (Transcutol^®^ P-employed) increased VST dissolution after 2 h by 2.5-fold and 1.6-fold, whereas the present formulation (GEL-employed) increased the dissolution by 6-fold and 2.3-fold, respectively. Thus, we expected that GEL could be used to replace Transcutol^®^ P while maintaining the homogeneous nanodispersion with high proficiency. This result might be due to the well-balanced composition of GEL, which has short, medium, and long chain fatty acids.

### 3.7. In Vivo Pharmacokinetic Evaluation 

**Figure 8:**
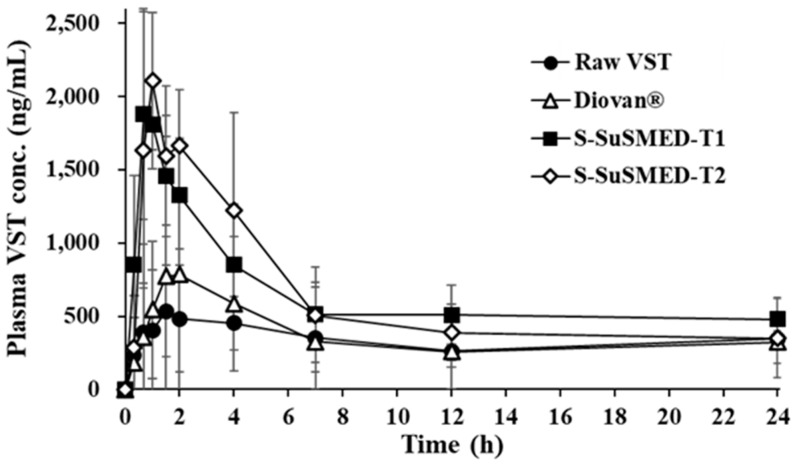
Plasma VST concentration profiles in rats after oral administrations of different formulations containing an equivalent dose of 10 mg/kg of VST. Values are presented as the mean ± SD (*n* = 8–12).

The pharmacokinetic study of VST in rats was evaluated after the oral administration of raw VST, Diovan^®^ (powder), S-SuSMED-T1 (powder), and S-SuSMED-T2 (powder). The plasma concentrations of VST were determined and plotted against time (Figure 8). The reference drug, Diovan^®^, showed similar pharmacokinetic behavior to raw VST throughout the whole time period, except for the peak time around 1.5–2 h. In comparison, the S-SuSMED tablets showed a sharp increase within 1 h, even though the plasma VST level of S-SuSMED-T2 was somewhat higher than that of S-SuSMED-T1. This rapidly increasing pattern, as reported in earlier literature [9,11,15], might be attributed to not only the VST dissolution enhancement of S-SuSMED in the acidic environment of the stomach but also the increased permeation of VST through the GI tract due to nano-sized emulsions [41].

The pharmacokinetic parameters of different samples are listed in Table 4. The *C*_max_ and AUC_0–24h_ values of Diovan^®^ were greater than those of raw VST, but were much lower than those of the S-SuSMED tablets, revealing a decreasing order of S-SuSMED-T1 > S-SuSMED-T2 > Diovan^®^ > raw VST for AUC_0–24h_ and S-SuSMED-T2 > S-SuSMED-T1 > Diovan^®^ > raw VST for *C*_max_. Based on the AUC_0–24h_ values, the RBA values of both the S-SuSMED tablets were approximately 1.8-fold greater than those of Diovan^®^. These results support the superiority of S-SuSMED tablets to Diovan^®^, indicating the potential of microemulsion-based formulations to enhance the oral absorption of VST [9]. In particular, GEL, a lipid excipient with an HLB value of 14, is a selectable vehicle not only for solubilizing poorly water-soluble drugs [21] but also for enhancing the absorption of low-BA drugs [42,43]. Moreover, the inhibitory effect of Tween^®^ 80 and GEL on P-gp-mediated efflux played a great role in enhancing the oral BA, as VST is a well-known P-gp substrate [44]. Although BCS class II drugs are highly permeable, the solubilizing and P-gp inhibitory effects of the SMEDDS component would be beneficial for BA improvement of these drugs. On the other hand, the *T*_max_ value of Diovan^®^ was smaller than those of raw VST, but was approximately 2-fold greater than those of S-SuSMED tablets. These results were in good agreement with the dissolution behavior described above. For BCS class II drugs, including VST, dissolution is a rate-limiting step for drug absorption [45]. Consequently, oral BA was increased by the S-SuSMED formulation: approximately 1.9-fold increase versus raw VST and 1.8-fold increase versus Diovan^®^. Compared with the earlier formulation (Transcutol^®^ P-employed), for which RBA was 2.2-fold greater than that of Diovan^®^ [15], the present formulation (GEL-employed) resulted in a less RBA increment. Although the RBA of S-SuSMED-T1 was a little higher than that of S-SuSMED-T2, there were no significant differences in all the pharmacokinetic parameters of both tablets. Therefore, we conclude that both of the S-SuSMED tablets would be useful for practical development in pharmaceutical industries in the future.

### 3.8. Correlations Between In Vitro DE and In Vivo Absorption

**Figure 9:**
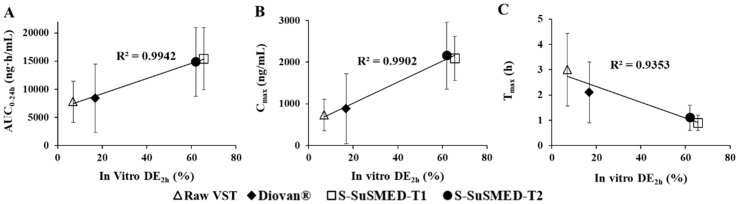
Correlations between in vitro DE_2h_ values and in vivo pharmacokinetic parameters of AUC_0–24_ (**A**), *C*_max_ (**B**), and *T*_max_ (**C**). Values are presented as the mean ± SD (*n* = 3). DE: dissolution efficiency.

The main role of in vitro–in vivo correlations (IVIVCs) is to provide surrogates for in vivo BA studies and assist biowaivers by validating the in vivo relevance to in vitro dissolution [45]. IVIVC comparisons can predict information about which formulations successfully enhance the BA. In this study, various samples, such as raw VST, Diovan^®^, S-SuSMED-T1, and S-SuSMED-T2, were compared. As an in vitro parameter, the advantage of DE is that it can be easily used to compare a large number of formulations [23]. Figure 9 shows the correlations of in vitro DE for 2 h (DE_2h_) with three in vivo pharmacokinetic parameters: AUC_0–24h_, *C*_max_, and *T*_max_. These in vivo parameters have been widely adopted for level C IVIVC study [46]. As shown in Figure 9A,B, in vitro DE_2h_ values exhibited good correlation with in vivo AUC_0–24h_ and *C*_max_ values, revealing an excellent linearity (*R*^2^ = 0.9942 and 0.9902, respectively). In addition, as shown in Figure 9C, *T*_max_ values were inversely correlated with in vitro DE_2h_ values in good linearity (*R*^2^ = 0.9353), indicating fast absorption of VST in S-SuSMED formulations. Therefore, we could suggest that enhanced in vitro dissolution of S-SuSMED-T1 and S-SuSMED-T2 is closely related to the improved in vivo absorption of VST. 

## 4. Conclusions

VST-loaded S-SuSMED formulations were successfully developed by employing GEL as a cosurfactant and HPC/FLO blend as solid carriers. The resultant S-SuSMED granules showed an excellent-to-fair flowability with enhanced dissolution, and they were further formulated into tablets by employing directly compressible excipients. Subsequently, S-SuSMED tablets were sufficiently stable for 6 months in an accelerated condition and showed enhanced in vitro dissolution. The in vivo oral BA of S-SuSMED tablets was approximately 1.8-fold greater than that of Diovan^®^, a commercial reference product. Therefore, VST-loaded S-SuSMED formulations might have great potential for practical development of a solidified dosage form, while enhancing the oral BA of VST. However, further investigations on allometric scaling and/or human pharmacokinetic evaluation are still needed.

## Figures and Tables

**Table 1:** Composition of various solidified SuSMED (S-SuSMED) granules and their reconstitution properties.

Granules	G1	G2	G3	G4	G5	G6
Composition (mg)						
SuSMED ^a^	190	190	190	190	190	190
HPC	19	57	95	133	171	209
FLO	247	209	171	133	95	57
Total	456	456	456	456	456	456
Reconstitution properties ^b^						
Droplet size (nm)	151.2 ± 22.3	146.6 ± 18.1	140.1 ± 13.8	137.5 ± 15.3	136.0 ± 11.4	139.2 ± 14.2
PDI	0.34 ± 0.06	0.32 ± 0.07	0.23 ± 0.04	0.24 ± 0.05	0.24 ± 0.02	0.27 ± 0.03

^a^ SuSMED was composed of VST (80 mg), Capmul MCM (10 mg), Tween 80 (30 mg), GEL (60 mg), and Poloxamer 407 (POL) (10 mg). ^b^ Values are presented as the mean ± SD (*n* = 10).

**Table 2:** Composition of the S-SuSMED tablets and their characteristics.

Tablets	S-SuSMED-T1	S-SuSMED-T2
Composition (mg)		
SuSMED ^a^	190	190
HPC	57	133
FLO	209	133
Kollidon^®^ 30	54.9	54.9
Primellose^®^	27.5	27.5
Magnesium stearate	11	11
Total	549.4	549.4
Characteristics ^b^		
Weight variation (mg)	549.1 ± 1.0	549.2 ± 1.0
Diameter (mm)	12.01 ± 0.01	12.03 ± 0.02
Thickness (mm)	4.83 ± 0.03	4.31 ± 0.02
Hardness (kP)	8.7 ± 0.1	8.6 ± 0.2
Friability (%)	0.1	0.6
Drug content (%)	97.1 ± 2.3	97.7 ± 1.9

^a^ SuSMED was composed of VST (80 mg), Capmul^®^ MCM (10 mg), Tween^®^ 80 (30 mg), GEL (60 mg), and POL (10 mg). ^b^ Values are presented as the mean ± SD (*n* = 10).

**Table 3:** Stability of various formulations in accelerated and long-term stability tests.

Formulations	Drug Content (%)			Total Unknown Impurities (%)		
	Initial	3 Months	6 Months	Initial	3 Months	6 Months
Accelerated stability test						
Diovan^®^	100.1 ± 1.5	100.8 ± 2.8	99.7 ± 1.3	BQL	BQL	0.06 ± 0.01
S-SuSMED-T1	99.8 ± 1.0	99.8 ± 0.8	97.6 ± 2.2	BQL	BQL	BQL
S-SuSMED-T2	99.7 ± 0.9	99.5 ± 1.2	98.6 ± 1.5	BQL	BQL	BQL
Long-term stability test						
Diovan^®^	100.1 ± 1.5	100.5 ± 1.9	99.9 ± 1.8	BQL	BQL	BQL
S-SuSMED-T1	99.8 ± 1.0	99.7 ± 1.7	98.8 ± 1.6	BQL	BQL	BQL
S-SuSMED-T2	99.7 ± 0.9	99.6 ± 1.2	99.2 ± 1.1	BQL	BQL	BQL

Values are presented as the mean ± SD (*n* = 6). BQL: below the quantification limit.

**Table 4:** Pharmacokinetic parameters of VST in various formulations in rats.

Parameters	Raw VST	Diovan^®^	S-SuSMED-T1	S-SuSMED-T2
AUC_0–24h_ (ng·h/mL)	7799.7 ± 3643.8	8381.2 ± 6086.1	15432.6 ± 5538.3	14867.6 ± 6092.9
*C*_max_ (ng/mL)	732.7 ± 372.9	880.0 ± 841.0	2092.6 ± 528.5	2156.0 ± 803.0
*T*_max_ (h)	3.0 ± 1.4	2.1 ± 1.2	0.9 ± 0.3	1.1 ± 0.5
Relative BA (%)				
versus raw VST	–	107.5	197.9	190.6
versus Diovan^®^	93.1	–	184.1	177.4

Values are presented as the mean ± SD (*n* = 8–12). AUC: area under the curve; BA: bioavailability.
